# Characterisation of interleukin-10 expression on different vascular structures in allergic nasal mucosa

**DOI:** 10.1186/2045-7022-4-2

**Published:** 2014-01-10

**Authors:** Barbara Muller, Danielle van Egmond, Esther JJ de Groot, Wytske J Fokkens, Cornelis M van Drunen

**Affiliations:** 1 Department of Otorhinolaryngology, AMC, Room L3-104-2, Meibergdreef 9, Amsterdam 1100 DD, The Netherlands

**Keywords:** Adhesion molecules, Allergic rhinitis, Blood vessels, Interleukin-10, Leukocyte trafficking, Nasal endothelium, Nasal allergen provocation

## Abstract

**Background:**

Interleukin-10 (IL-10) is a negative regulator of immune responses and was previously shown to be expressed by human nasal endothelial cells, while the adhesion molecule MECA-79 plays a role in trans-endothelial migration of immune competent cells. In this study we investigate the relationship between endothelial IL-10 and MECA-79 expression to address the question whether immune competent cells could be affected at the mucosal entry site.

**Methods:**

Nasal turbinate biopsies were taken from house dust mite allergic patients, before and after nasal allergen provocation. Subsequent slides of biopsies were stained for IL10, MECA-79, CD34, and IL10-Receptor. Capillaries, arteries/veins, and sinusoids were evaluated separately.

**Results:**

90% of sinusoids are IL-10 positive and all sinusoids are negative for MECA-79, while 4.8% of capillaries are positive for IL-10, and 2.2% are positive for MECA-79. Although about 47% of arteries/veins are positive for IL-10 and 57.1% are positive for MECA-79, only about 20% are positive for both markers. Furthermore, we showed that the myo-fibroblasts surrounding all sinusoids stain positive for IL10R.

**Conclusions:**

IL10 expression on vascular structures is not related to MECA expression for sinusoids and capillaries and only partly related on arteries/veins, however sinusoidal endothelial IL10 expression is always seen in combination with IL-10R expression of sinusoidal myo-fibroblasts.

## Background

Allergic rhinitis is a disease with a severe impact. As many as 10-25% of the population in Western societies suffers from allergic rhinitis and its prevalence of allergic rhinitis continues to increase [1]. Rhinitis can significantly decrease quality of life and creates significant direct and indirect costs [1-3]. Although the pathogenesis of allergic rhinitis has been studied extensively, many questions remain unanswered. Within our research into immune regulatory processes in the nasal mucosa, we have been focusing on structural cells in the human nasal mucosa. The immune function of epithelial cells as well as endothelial cells have been addressed in previous reports [4-8]. We previously found expression of Interleukin-10 (IL-10) on the endothelial cells in the nasal mucosa of allergic subjects. This endothelial IL-10 expression appeared to have a strong inverse correlation with the induced symptoms of allergic rhinitis [8]. In our previous study we did not explore potential mechanisms by which endothelial IL-10 expression could influence the level of allergic symptoms. Given that vascular endothelium form a gateway for inflammatory cells into the local mucosa, one possible explanation is that during endothelial transmigration the pro-inflammatory characteristics of cells entering the mucosa are dampened through the local exposure to endothelial IL-10. In order to explore this option we investigated what vascular structures could play a role in trans-migration by identifying the distribution of MECA-79, a adhesion molecule involved in transmigration, on nasal endothelium.

The nasal mucosa is characterized by a complex micro vascular anatomy. It consists of a dense subepithelial network of capillaries, a system of capacitance vessels or sinusoids and arteriovenous anastomoses; the arteries/veins. Vascular activities are involved both in the mucosal defence and physiological functions of the nose, thereby also contributing to inflammatory airway diseases such as allergic rhinitis [9]. Nasal obstruction is a crucial symptom in allergic rhinitis and it is primarily a vascular phenomenon caused by distension of the sinusoids with blood [10]. Furthermore, by opening the gaps in the intercellular junctions between the endothelial cells, extravasation of plasma takes places, which ultimately contributes to rhinorrhoea formation and swelling of the nasal mucosa. Lastly, the influx of inflammatory cells and mediators of the early and the late inflammatory phase from blood to the local tissue contributes to nasal blockage, rhinorrhoea, sneezing, and pruritus [11].

We investigated the three morphologically different endothelial structures vessels separately. Firstly, the sinusoids are specialized structures that may expand and contract thanks to smooth muscle actin (SMA) positive (myo)fibroblasts that line these structures. Their capacitance volume is under neural regulation and may also be influenced by humoral factors, allowing for direct action on the vasculature, or indirectly via sensory neural stimulation [12].

Secondly, the blood vessels are the main avenue by which inflammatory cells reach the nasal mucosa. Given the role of IL-10 in regulating the activity of mast cells [13-16] dendritic cells [17,18] and T cells [19,20], the transendothelial migration of inflammatory cells could present an important checkpoint.

Thirdly, capillaries’ main function of local oxygen supply would at best only indirectly contribute to an allergic response, albeit that, like for all types of blood vessels, vascular leakage from capillaries will contribute to mucosal swelling and rhinorrhoea.

In this manuscript we will focus on potential mechanisms by which endothelial IL-10 expression may affect clinical symptoms. To this extend we looked into the expression of IL-10 and the IL-10 receptor on the different endothelial structures in the nasal mucosa. In order to look into the effect on cell-trafficking, we identified the main cellular entry sites for inflammatory cells in the nasal mucosa by using the MECA-79 antibody which is directed against ligands of L-selectin, We aimed to investigate how these entry sites relate to endothelial IL-10 expression, at baseline as well as after nasal provocation.

## Methods

In a previous study [21] we collected nasal biopsies of twenty-one persistent rhinitis patients (median age 42, range 17-63) with positive house dust mite (HDM) skin-prick tests or radioallergosorbent tests (RAST), and with symptoms for more than 1 year. Patients with positive skin-prick test for other allergens besides dust mite were not excluded as long as these sensitizations were not clinically relevant and none of the patients used any symptomatic medication in the four weeks prior to the study. The medical ethics committee of the Erasmus Medical Centre approved of this study (MEC 188.581/2000/30) and all patients gave their written informed consent.

On the first day, a baseline mucosa biopsy was obtained. On day 6, patients received bilateral nasal provocation using a nasal spray delivering a fixed volume of 0.089 ml of 1000 biological units (BU)/ml HDM (total amount of 2 × 89 BU) [*Dermatophagoides pteronyssinus* ALK-Abellò, Nieuwegein, the Netherlands]. On day 7 (24 hours after the provocation), patients re-entered the clinic and a second nasal biopsy was obtained. All visits took place between February and May 2000, outside the Dutch grass pollen season. Neither vehicle provocation nor local anaesthesia led to the induction of clinical symptoms [21]. All biopsy specimens of nasal mucosa were taken from the inferior turbinate by the same investigator using the method described earlier [22].

### Immunohistochemistry

Specimens were snap frozen and stored for immunohistochemistry. Briefly, each tissue specimen was cut into serial, 5-μm-thick sections on a Micron HM 560 Frigocut and transferred onto microscope slides (Sigma Chemical Co. St. Louis, MO, USA) coated with APES (amino-phosphate-ethylsilane), dried and stored at -80°. For staining according to manufactures procedures, slides were brought up to room temperature, air dried, and fixed in acetone for 10 min at room temperature. The slides were then rinsed in phosphate buffer saline (PBS; pH 7.8) and placed in a semi-automatic strainer [Sequenza Shandon, Sewickley, PA, USA] and incubated with 10% (v/v) normal goat serum (NGS) [CLB, Amsterdam, the Netherlands] for 10 minutes. To block endogenous avidine and biotin, antibodies were diluted in 1% (v/v) blocking reagent [Roche, Basel, Switserland]. The sections were then incubated for 60 minutes at room temperature with the primary antibody. Mouse anti-human monoclonal antibodies directed against functionally active L-selectins (CD62L) as detected with anti-MECA-79 (dilution 1:100) [BD Pharmigen] and against endothelial cells (anti-CD34 dilution 1:400) [MONOSAN/Sanbio, Uden, the Netherlands] were used, or appropriate IgG isotype control (ABCAM, Cambridge, UK) at room temperature. The sections were then rinsed with PBS for 5 minutes and incubated for 30 minutes with a biotinylated goat anti-mouse (1:50) immunoglobulin antiserum [Biogenics, Klinipath, Duiven, the Netherlands] at room temperature. Subsequently, the sections were rinsed with PBS for 5 minutes and incubated for 30 minutes with alkaline-phosphatase conjugated goat anti-biotin, and New Fuchsine substrate for 20 minutes.

Staining against IL-10 receptor was performed with poly power vision-mouse-AP against IL-10R (dilution 1:50) [Klinipath, Duiven, the Netherlands] according to manufacture’s instructions. Slides were incubated for 30 minutes. The sections were then rinsed with PBS for 5 minutes and incubated for 30 minutes with PV-mouse-AP at room temperature. The sections were then rinsed with PBS for 5 minutes and incubated with New Fuchsine for 20 minutes. Subsequently, the slides were incubated with haematoxylin for 10 min.

Staining with anti IL-10 (dilution 1:150) was performed using tyramine signal amplification (TSA). After incubation with biotinylated goat anti-mouse Ig serum, endogenous peroxidise was blocked using 0.2% (w/v) hydrogen peroxide and 50% (v/v) methanol in PBS. Slides were then incubated with streptavidin conjugated peroxidise [NEN, Boston, MA, USA] for 30 minutes, biotinyl tyramide in Tris-HCl buffer for 10 minutes for amplification of the signal, alkaline-phosphatase conjugated goat anti-biotin, and New Fuchsine substrate for 20 minutes [23].

### Microscopically assessment of histochemical staining

All slides were evaluated by two observers who were blinded from the clinical outcomes and time point of provocation. The number of positive and negative vessels was determined per square millimetre. The capillaries, arteries / veins, and sinusoids were distinguished on the basis of histology, as well as the CD-34 [24].

### Statistical analysis

We used the Mann-Whitney test to assess statistical differences between medians of different groups. P-values < 0.05 were considered statistically significant.

## Results

### Distinct endothelial expression of IL-10 on different types of blood vessels

**Figure 1:**
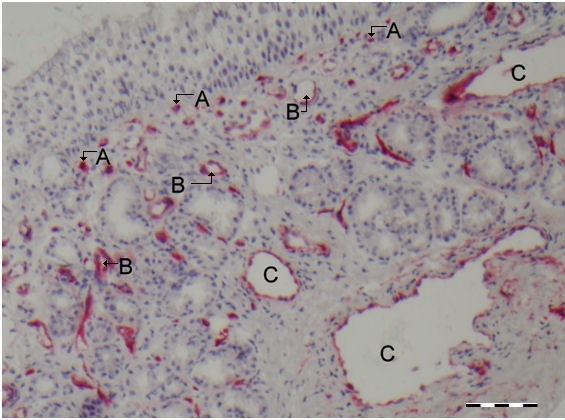
**A representative cross section of the nasal mucosa with vascular structures stained with CD34.** Indicated are capillaries **(A)**, arteries/veins **(B)**, and sinusoids **(C)**. Scale bare = 100 μm.

**Figure 2:**
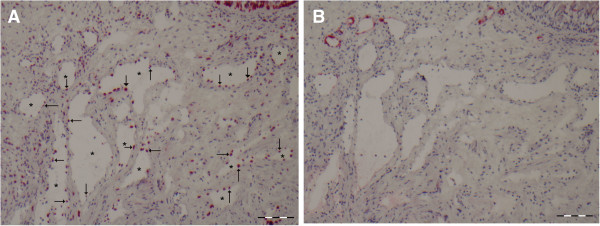
**A representative cross section of the nasal mucosa stained with IL-10 or MECA.** In panel **A** the arrows and * indicate sinusoids staining positive for IL-10, while panel **B** shows the absence of MECA staining on sinusoids. (scale bare = 100 μm).

**Figure 3:**
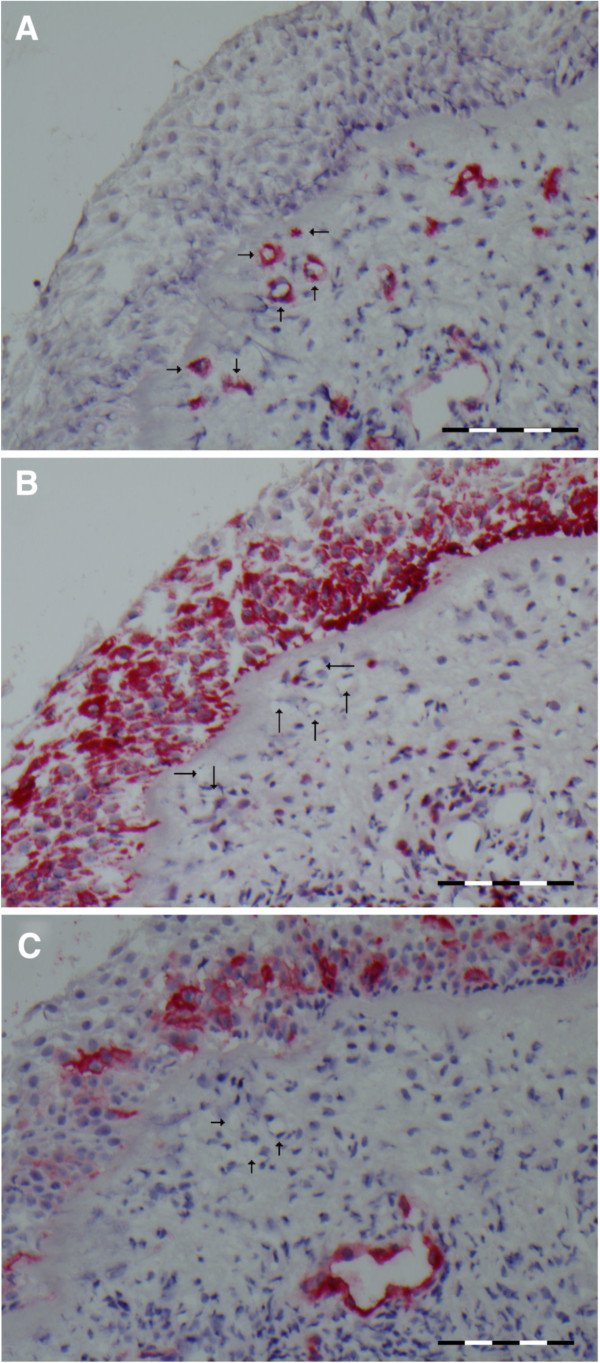
**A representative cross section of the nasal mucosa showing capillaries (arrows) staining positive for CD34 (A), negative for IL-10 (B), and negative for MECA (C).** Scale bare = 200 μm.

**Figure 4:**
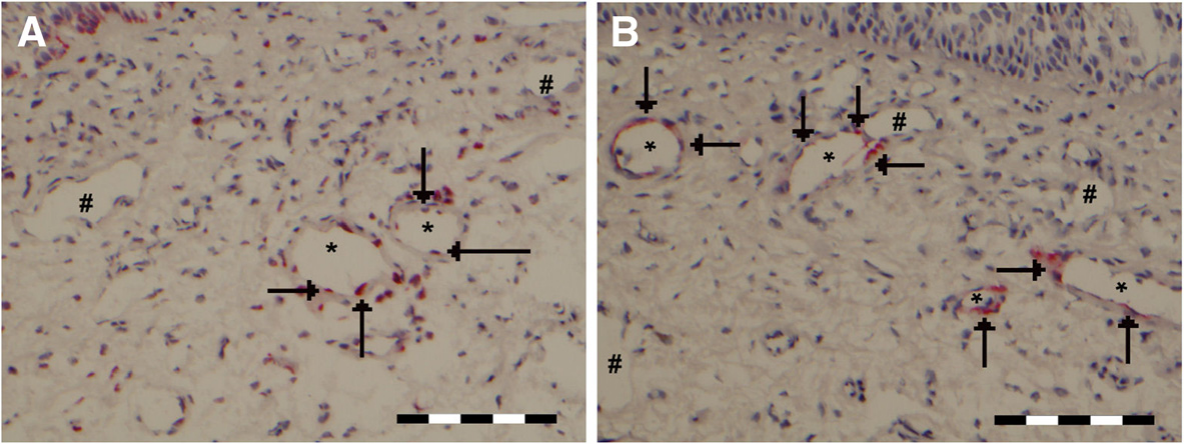
A representative cross section of the nasal mucosa showing (A) IL-10 positive (arrows and *) and negative (#) arteries/veins, and (B) MECA positive (arrows and *) and negative (#) arteries/veins. Scale bare = 100 μm.

In this analysis we consider the different types of endothelial structures in the nasal submucosa. To detect the vessels, we used the general blood vessel specific marker CD34 (Figure 1). We differentiated between capillaries (marked with an ‘A’ in Figure 1), arteries/veins (marked with a ‘B’ in Figure 1), and sinusoids (marked with a ‘C’ in Figure 1), based on morphology. On a successive slide we subsequently determined IL-10 expression and enumerated IL-10 positivity for each of the three types of blood vessels. A representative example of IL-10 positive sinusoids is shown in Figure 2A. Nearly all sinusoids are positive for IL-10 (median 90.9%, range 80%- 100%). These numbers are in contrast with the low level of IL-10 expression on capillaries (median 4.8%, range 0%-50%). Comparing Figure 3A with Figure 3B (subsequent slides) shows the presence of CD34 positive capillaries that are IL-10 negative. The arteries/veins are situated in the middle of this spectrum (Figure 4A) with about half of the vessels staining positive for IL-10 (median 46.7%, range 17%-76 %).

### Sinusoids and capillaries do not act as entry sites for inflammatory cells

Next we investigated which types, or parts, of vessels could act as entry site for inflammatory cells by determining the expression of activated L- selectin ligand (MECA-79), which is required for transendothelial migration. Under steady state conditions, hardly any MECA expression could be detected on sinusoids (median 0 %, range 0%-12% (Figure 2B) and capillaries (median 2.2%, range 0%-6%). Given the high percentage of IL-10 positive sinusoids, the few sinusoids that were MECA positive were also IL-10 positive. For capillaries (Figure 3B, 3C) there was no obvious relationship between the few capillaries that were IL-10 or MECA positive.

### Arteries and veins are the main entry sites for inflammatory cells

**Figure 5:**
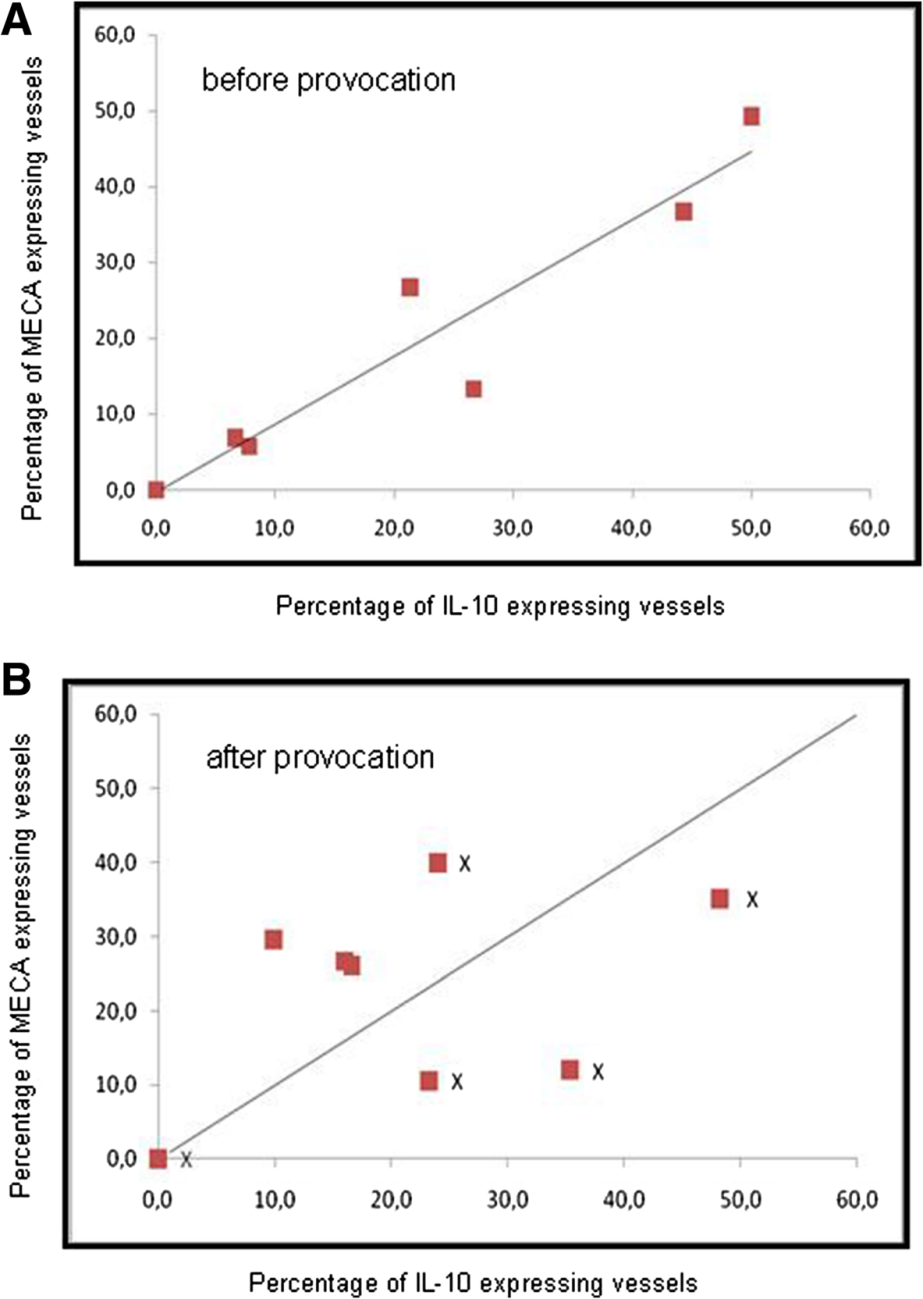
Scatter plot of IL-10 and MECA expression on arteries/veins before (A) and after (B) nasal provocation.

MECA expression appeared to be limited to arteries and veins within the nasal mucosa (Figure 4B). At baseline, slightly over half (median 57.1%, range 0 – 100) of these vessels are MECA positive and thus would be able to facilitate transendothelial migration based on the presence of the sulphated and glycosylated L-selectin epitopes. There does not seem to be a direct correlation between MECA expression and IL-10 expression on these vessels. About half of the vessels (median 48.3%, range 17 – 78%) stain positive for IL-10, while the median percentage for MECA and IL-10 double positive vessel is 13.3% (range 0 – 49%). The observed median percentage (13.3%), as well as the observed mean percentage (19.8%) of double positive vessels is close to a predicted median value (19.7%) and mean percentage (17.0%) when assuming random distribution of MECA and IL-10 positivity. Also when the data is plotted per individual the percentages of vessels positive for the separate markers predict the percentage of vessels that are positive for both IL-10 and MECA (Figure 5A).

### IL-10 expression of sinusoids could be related to vasodilatation/vasoconstriction

**Figure 6:**
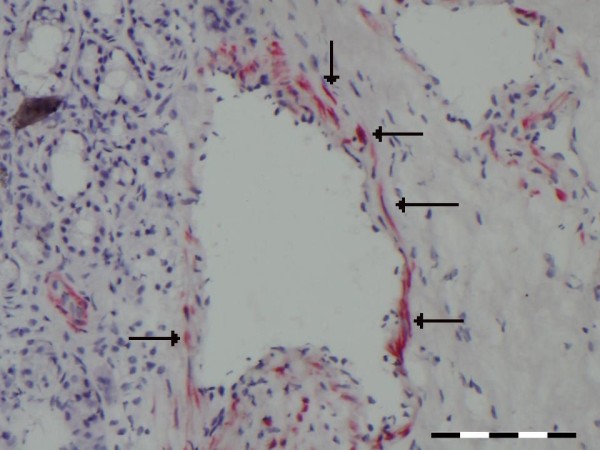
**A representative cross section of the nasal mucosa showing myo-fibroblasts around the sinusoids staining positive for the IL-10 receptor (arrows).** Scale bare = 100 μm.

The absence of activated L-selectins on IL-10 positive sinusoids precludes a role of endothelial IL-10 in the modulation of transmigrating inflammatory cells. Thus, the IL-10 produced by the endothelial cells of positive sinusoids could have its effect on allergic symptoms by influencing other cells, possibly cells in the vicinity of the sinusoids. Here we show that the myo-fibroblasts that line the sinusoids express the IL-10R (Figure 6).

### Nasal provocation: a complicated effect on the relationship between MECA and IL-10

**Figure 7:**
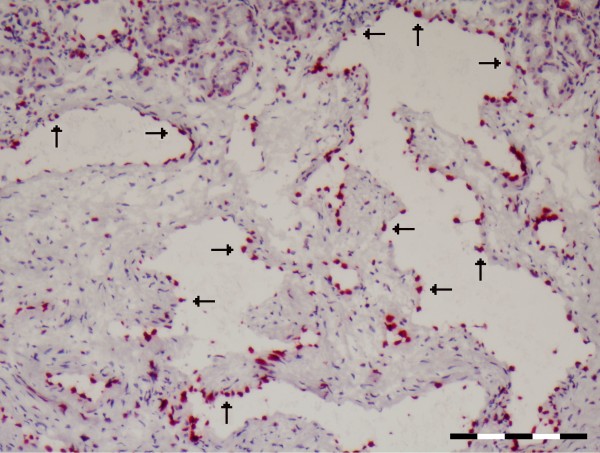
**A representative cross section of the nasal mucosa vessels after allergen provocation showing extensive IL-10 positivity in sinusoids (arrows).** Scale bare = 200 μm.

Nasal provocation seems to have a limited effect on the general allocation of both IL-10 and MECA epitopes among the different vessels (Table 1, and Figure 7). Although there are some minor shifts in the percentages of the different vascular structures staining positive for either IL-10 or MECA, only the increase of IL-10 positive staining sinusoids is statistically significant.

**Table 1:** Percentages of IL-10 and MECA positivity for different vascular structures before and after nasal provocation

	**IL-10% before provocation median (range)**	**IL-10% after provocation median (range)**	**MECA% before provocation median (range)**	**MECA% after provocation median (range)**
**Sinusoids**	90.6 (80–100)	100 (88–100)	0.0 (0–12)	2.0 (0–20)
**Vessels**	46.7 (17–76)	37.4 (0–54)	57.1 (0–100)	53.9 (25–89)
**Capillaries**	4.8 (0–50)	5.2 (0–14)	2.2 (0–6)	2.7 (0–25)

However, nasal provocation does seem to influence the relationship between IL-10 and MECA on arteries/veins. Where prior to provocation vessels that stain double positive is predicted by the individual percentages that stain positive for IL-10 or MECA (Figure 5A), after provocation the distribution of double positive vessels seems less random (Figure 5B). Here the patients separate into two groups: a group with individuals where fewer vessels than predicted stain positive for both markers (below the line) and a group with individuals where more vessels than predicted stain positive (above the line) for both markers. We tried to correlate this picture to the amount of symptoms we previously registered after provocation [21]. We were not able to establish a clear relationship with the level of symptoms induced by the nasal provocation, albeit that 3 out of the 5 individuals with the highest level of symptoms (marked with an ‘X’ in Figure 5B) display a lower than expected level of MECA and IL-10 double positive staining blood vessels.

## Discussion

For cell influx through vessels the expression of adhesion molecules on these vessels is required. Normally, the expression of L-selectin ligands is restricted to high endothelial venules (HEVs) in lymphoid tissues. However, in diseases characterized by chronic inflammation such as asthma, Crohn’s disease and rheumatoid arthritis induction of L-selectin ligands is seen in postcapillary endothelial cells in tissues other than lymphatic tissue [25,26]. It appears that the expression level of L-selectin ligands (as identified with MECA-79 expression) correlates with the extent of tissue eosinophilia and severity of inflammation in patients with chronic rhinosinusitis [26,27]. In accordance with the literature on L-selectin ligand expression in chronic inflammatory diseases, the overall expression of MECA-79 in inferior turbinate specimens of our allergic subjects appeared to be higher compared with what was previously found on inferior turbinate specimens of healthy controls [27]. The L-selectin ligand expression in the allergic turbinates is also higher than the expression that was previously found in maxillary sinus mucosa of chronic rhinosinusitis patients [26]. The nasal mucosa of house dust mite allergic rhinitis patients must be chronically inflamed. Still, if L-selectin ligand expression in these tissues would be related to inflammation, one would expect this expression to be up regulated after successful provocation. However, we did not find this. L-selectin expression on these vessels could reach a ‘plateau’ in result to the constant exposure to allergens which house dust mite allergic patients suffer from. This matter needs further research and it would be interesting to compare this expression pattern to that in patients that are solely allergic to grass pollen (in- and out of season).

To our knowledge, this is the first detailed report on MECA- and IL-10 distribution among the different vascular structures in the nasal mucosa. We found that the sinusoids are mostly negative for MECA. This expression pattern suggests that, at the site of the sinusoids, IL-10 cannot play a role by influencing the activity of cells entering the tissue influx. The presence of IL-10R positive (myo)-fibroblast around the sinusoids suggests that we must consider a direct affect on the sinusoids. This staining pattern suggests the existence of a ‘functional unit’ of IL-10 expressing sinusoidal endothelial cells and IL-10 receptor expressing myofibroblasts around these sinusoids. Such functional unit could theoretically influence vasoconstriction and relaxation of these sinusoids. Interestingly, it was shown that IL-10, in addition to its anti-inflammatory activity, influences the contractibility of (myo)fibroblasts [28]. Because the nasal tissues are so highly vascular, vascular changes can lead to significant nasal obstruction. In allergic patients, a shift in allergic symptoms could be reflected on the IL-10 expression of the sinusoids and vice versa.

In the current study, we saw a significant rise in sinusoidal IL-10 expression after provocation. This could be in line with the observation that IL-10 has a direct inhibitory effect on smooth muscle cells [29] which would result in congestion and a rise in symptoms. The molecular mechanism that could play a role in the regulation of IL-10 in endothelial/structural cells remains yet unclear, as most reports concerning IL-10 and endothelium concerns the effect of IL-10 on endothelium [30,31] rather than its expression by endothelium. It remains to be explored whether triggers that previously have been shown to affect IL-10 expression, like Fc-receptor activation in mouse dendritic cells DC [32] or histamine exposure of human dendritic cells [33], also plays a role in human nasal endothelial cells.

Capillaries do not seem to play a role in the inverse relationship between endothelial IL-10 expression and clinical symptoms, as these structures are hardly ever IL-10 (4.8%) or MECA (2.2%) positive. The most complicated relationship we observed between IL-10 and MECA is for the remaining blood vessels comprised of arteries and veins. Both markers are expressed at about 50% of the vessels. However these are not the same vessels. Detailed analysis of arteries/veins suggests that IL-10 and MECA have an independent expression pattern, were the number of double positive vessels in a given patient can accurately be predicted on the basis of the individual expression pattern of IL-10 and MECA in that same patient. Even if we would consider this observation to be representative of a potentially dynamic inflammatory process, this would still imply that in a substantial number of blood vessels the endothelial IL-10 expression would be able to influence the biological activity of inflammatory cells that migrate into the tissue. Although potentially interesting we have not explored whether the 50% of vessels that stain positive for either IL-10 or MECA could represent just arteries or veins.

While elaborating on the function of IL-10 localised on the different vessels we can think of two possible ways in which IL-10 could work. Given the inhibitory effect of IL-10 on leukocytes [34], the first way would be to influence these cells in the blood stream or when they that are passing through the endothelium into the tissue. A clue that this aspect could play a role comes from the observation that the inverse relationship between endothelial IL-10 expression and clinical symptoms (as described previously [8]) appeared to be the strongest for the symptoms of the late phase response. The late phase response is dominated by the influx of inflammatory cells [12,35]. The second way that IL-10 could influence clinical symptoms is a localized action of IL-10 on the blood vessels themselves. As IL-10 has been described to act on contractibility of vessels [28], it could influence vascular activities such as contraction and distension of vessels and opening of the gaps in the intracellular junctions since there is IL-10 receptor expression around the vessels. We strongly believe that endothelial structures, just like the endothelium, play an important regulatory role in the nasal mucosa. Therefore, we plan to further investigate the effect of IL-10 on the endothelium in vitro in future studies. Furthermore, we aim to find a way to differentiate between the arteries and the veins within the nasal mucosa in order to unravel distinct properties of these structures.

## Conclusions

In the present study, on the basis of immunohistochemical findings, elaborations were made on two possible ways in which endothelial IL-10 expression could play a role in symptoms of allergic rhinitis. We hypothesize that both a direct effect of sinusoidal IL-10 expression on the surrounding (myo)fibroblasts and an indirect effect of IL-10 on the biological activity of migrating inflammatory cells is likely. Our approach also revealed that although blood vessels form a continuum that each of the type of vessels (sinusoids, capillaries and arteries/veins) has an a unique expression pattern in regards to IL-10 and MECA, and that even within the latter group of arteries and veins local differences occur. This work contributes to our understanding of local regulation of inflammatory processes.

## Competing interests

WF and CvD have received private and public funding for basic research and clinical studies from GSK, Allergopharma, Stallergen, ALK, NWO, FWO, and the European union. The other authors have no conflict of interest.

## Authors’ contributions

BM analysed/interpreted the data and drafted the manuscript, DvE and EdG preformed the immunohistochemistry and contributed the manuscript, while WF and CvD designed the study, interpreted the data, and contributed to the manuscript. All authors have approved the final version of the manuscript.
